# Insight into the Self-Assembling Properties of Peptergents: A Molecular Dynamics Simulation Study

**DOI:** 10.3390/ijms19092772

**Published:** 2018-09-14

**Authors:** Jean Marc Crowet, Mehmet Nail Nasir, Nicolas Dony, Antoine Deschamps, Vincent Stroobant, Pierre Morsomme, Magali Deleu, Patrice Soumillion, Laurence Lins

**Affiliations:** 1 Laboratoire de Biophysique Moléculaire aux Interfaces, Gembloux Agro-Bio Tech, University of Liège, Passage des déportés 2, 5030 Gembloux, Belgium; jeanmarccrowet@gmail.com (J.M.C.); mn.nasir@gmail.com (M.N.N.); n.dony@skynet.be (N.D.); magali.deleu@uliege.be (M.D.); 2 Institut des Sciences de la Vie, Université catholique de Louvain, 4-5 Place Croix du Sud, 1348 Louvain-la-Neuve, Belgium; antoine.deschamps@outlook.com (A.D.); pierre.morsomme@uclouvain.be (P.M.); patrice.soumillion@uclouvain.be (P.S.); 3 Ludwig Institute for Cancer Research, de Duve Institute and Université Catholique de Louvain, 75 Avenue Hippocrate, 1200 Brussels, Belgium; vincent.stroobant@bru.licr.org

**Keywords:** peptide, self-assembly, molecular dynamic simulations, peptergent

## Abstract

By manipulating the various physicochemical properties of amino acids, the design of peptides with specific self-assembling properties has been emerging for more than a decade. In this context, short peptides possessing detergent properties (so-called “peptergents”) have been developed to self-assemble into well-ordered nanostructures that can stabilize membrane proteins for crystallization. In this study, the peptide with “peptergency” properties, called ADA8 and extensively described by Tao et al., is studied by molecular dynamic simulations for its self-assembling properties in different conditions. In water, it spontaneously forms beta sheets with a β barrel-like structure. We next simulated the interaction of this peptide with a membrane protein, the bacteriorhodopsin, in the presence or absence of a micelle of dodecylphosphocholine. According to the literature, the peptergent ADA8 is thought to generate a belt of β structures around the hydrophobic helical domain that could help stabilize purified membrane proteins. Molecular dynamic simulations are here used to image this mechanism and provide further molecular details for the replacement of detergent molecules around the protein. In addition, we generalized this behavior by designing an amphipathic peptide with beta propensity, which was called ABZ12. Both peptides are able to surround the membrane protein and displace surfactant molecules. To our best knowledge, this is the first molecular mechanism proposed for “peptergency”.

## 1. Introduction

By manipulating the various physicochemical properties of amino acids, the design of peptides with specific self-assembling properties has been emerging for some years [1]. Due to their biocompatibility and chemical diversity, peptides are an attractive platform for the design of various nanostructures, such as nanotubes, vesicles, fibers, micelles or rod–coil structures, that have potential applications in drug delivery, tissue engineering, or surfactants [2,3]. Depending on the sequence and environment, peptides can self-assemble into ordered structures constrained by non-covalent interactions, such as electrostatic interactions, hydrophobic interactions, van der Waals interactions, hydrogen bonds, and π-stacking. Particularly, amphiphilic peptides have shown their ability to self-assemble into a range of nanostructures [3] and behave in some respects similar to conventional amphiphilic molecules such as surfactants, detergents, and lipids. Amphipathicity can arise either from peptides containing polar and non-polar residues distributed regularly along the peptide [4], or from alkyl chains linked to a hydrophilic peptide.

In that way, short peptides possessing detergent properties (so-called “peptergents”) have been developed in the last decade to self-assemble into well-ordered nanostructures that can stabilize membrane proteins for crystallization [5]. Three main classes are described in the literature: amphipathic helical peptides [6], lipopeptides [7,8,9], and short lipid-like peptides [10,11,12]. From a conformational point of view, some of these peptides can adopt α helical or extended β sheet structures during their self-assembly. Recently, Zhang’s team engineered a β-sheet peptide that was able to self-assemble and sequester integral membrane proteins (IMPs) [13]. The peptide is amphipathic, alternating hydrophobic and hydrophilic residues. It is also methylated at some amino groups, and is grafted with two alkyl chains. It is proposed that the peptide is able to associate with IMPs in a β barrel-like configuration.

There are many detergents that are available for the solubilization and crystallization of membrane proteins [14]. However, these detergents need to stabilize the native structure of the protein in order to maintain its function and avoid aggregation. Finding the optimal detergents for the protein studied requires wide screening and depends on the application [12]. New ones are required, and some peptergents have shown better stabilizing properties than commonly used alkyl chain surfactants [15]. Even though experimental evidence is available concerning the relative efficacy of peptergents in solubilizing and stabilizing IMPs [10,11,13,16], little is known about the molecular mechanisms involved in their interactions with proteins.

**Figure 1:**
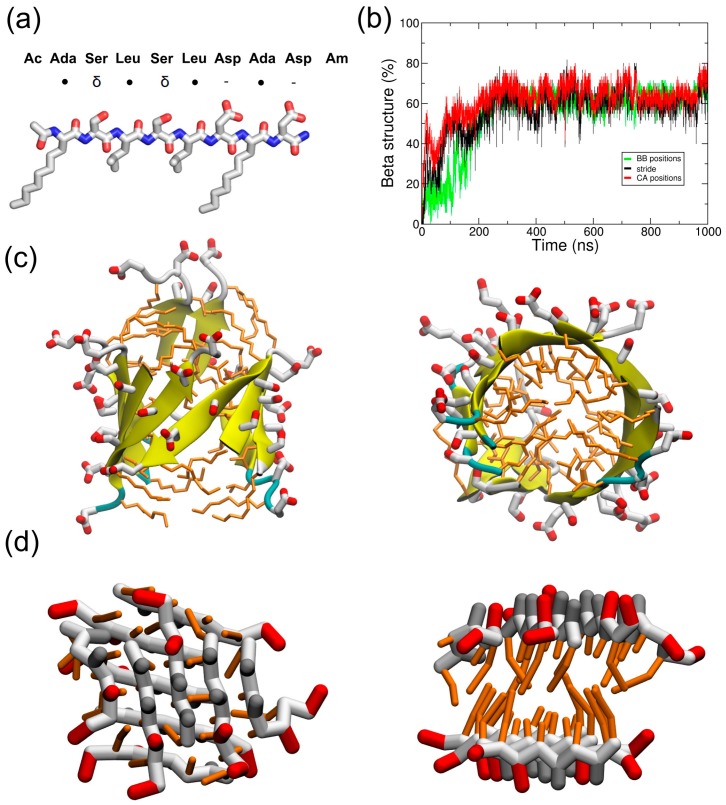
ADA8 peptides structure in water. (**a**) Representation of the ADA8 peptide. ●, δ, and - represent hydrophobic, polar, and negatively charged residues, respectively. (**b**) Percentage of beta conformation. The structure is assigned by stride in atomistic (AT) (black) and by using the following parameter in coarse-grained (CG) representations: a dihedral angle greater than 100° for four following CA atoms and two other following CA atoms within 6 Å. The red and green curves correspond to this parameter for AT and CG simulations. Conformations at the end of simulations in atomistic and coarse grained representations are in panels (**c**,**d**), respectively; the right panels are an upper view of the left panels. AT beta sheets are in yellow in the AT representations. Polar, negatively charged and hydrophobic CG residues are represented in gray, red and orange, respectively.

In this work, we studied the self-assembling properties of a known peptide called ADA8 [13] (Figure 1a) using molecular dynamic (MD) simulations. In water, this peptide is able to self-assemble as a beta barrel-like structure. In the presence of an integral membrane protein, the peptide forms a beta belt around the protein with and without surfactant molecules. To the best of our knowledge, this is the first time that a molecular mechanism is proposed by MD for “peptergency”. Our calculation approach should further serve as a predicting tool for the design of beta peptergents with diverse amphipathic properties, as suggested for a de novo designed peptide, ABZ12.

## 2. Results

### 2.1. Simulations in Water

Ten ADA8 peptides were simulated in water in atomistic (AT) (1 µs) and coarse-grained (CG) (10 µs) representations to follow their self-assembly (Figure 1). The black curve of Figure 1b shows a rapid increase in the beta structure during AT simulations in water, and the beta sheets that are formed can be seen in Figure 1c and Figure S1a. These structures are similar to beta barrels, with the peptide adopting amphiphilic beta strand conformations with the hydrophobic residues facing the inside of the barrel. The strands can be parallel or antiparallel. The self-assembly has also been simulated in a CG representation, since longer time scales can be achieved. At the end of the CG simulation, we observed two amphiphilic beta sheets facing each other, with the hydrophobic residues buried between the sheets (Figure 1d and Figure S1b). Polar interactions between sidechains as well as backbone interactions between the strands are also noticed. To estimate the formation of beta structures, a parameter based on Cα or backbone beads positions has been used (see Section 4). As observed in AT simulations, the peptides are able to adopt a beta conformation along the simulation, as assessed by the green curve in Figure 1b and the molecular assembly in Figure 1d and Figure S1b. However, the beta sheet twist observed in atomistic simulations is not reproduced by the CG model and leads to the formation of two facing beta sheets instead of a beta barrel. It is worth noting that the MARTINI force field is, in principle, not designed to sample native conformations [17], especially for beta sheet structures, since there is no hydrogen bond representation. In the literature, several fibril-forming peptides have been studied using MARTINI [18,19], such as the aggregation of the Apo C-II amyloid peptide or the assembly of the protofibrils of amylin. However, beta sheet formation was not observed for the Apo C-II peptide, and the beta conformation of amylin protofibrils was restrained before elongation could occur. Nevertheless, Seo et al. observed beta sheet formation with MARTINI, but they modified the backbone potentials to reproduce the structural properties derived from atomistic simulations [20]. Here, we observed the appearance of beta sheets without any modification of the force field; this is mainly due to the beta amphipathic nature of the ADA8 peptide. Some differences between CG and AT beta sheets were nevertheless observed. CG strands in beta sheets are shifted by one residue compared to atomistic beta sheets (Figure 1d). In the latter, the relative positions of the beta strands are defined by hydrogen bonding, and the side chains on both sheet sides align. In contrast, in CG, the attraction comes from the backbone (BB) beads, and the shifted position minimizes the overall BB bead distance between strands. The question of the backbone representation in MARTINI was discussed by Marrink et al. in 2013; a perspective evolution of the force field would be to add charged beads to the backbone in order to reproduce the structural preferences of proteins [21].

To assess the stability of the beta structure formed, the structure represented on Figure 1d has been transformed to an atomistic resolution and further simulated for 100 ns. This simulation shows a rapid reorganization of the beta sheets to form a beta barrel (Figure S2). Globally, for all of the simulations, the peptides are able to form beta structures, which agrees with the literature [13].

### 2.2. Coarse-Grained Simulation of the ADA8 Peptide in the Presence of a Membrane Protein

**Figure 2:**
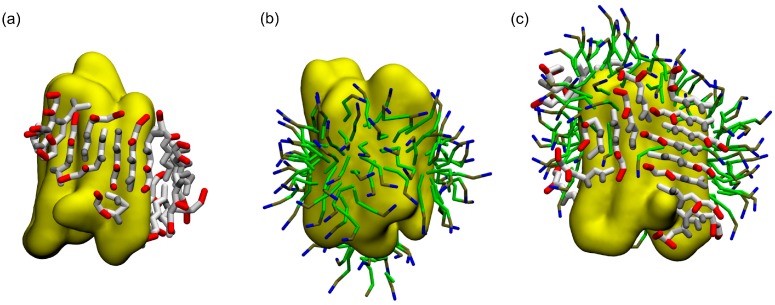
ADA8 peptide organization on the surface of the membrane protein. The structures show 20 peptides at the end of the CG simulations when the protein (in yellow) is alone (**a**) or covered by dodecylphosphocholine (DPC) (**c**); the protein surrounded by DPC is shown (**b**); and this structure was used as a starting point before the addition of the peptides (**c**). Polar, negatively charged and hydrophobic CG residues are represented in gray, red and orange, respectively. Acyl chain, phosphate and choline groups of DPC are in green, brown and blue, respectively.

**Figure 3:**
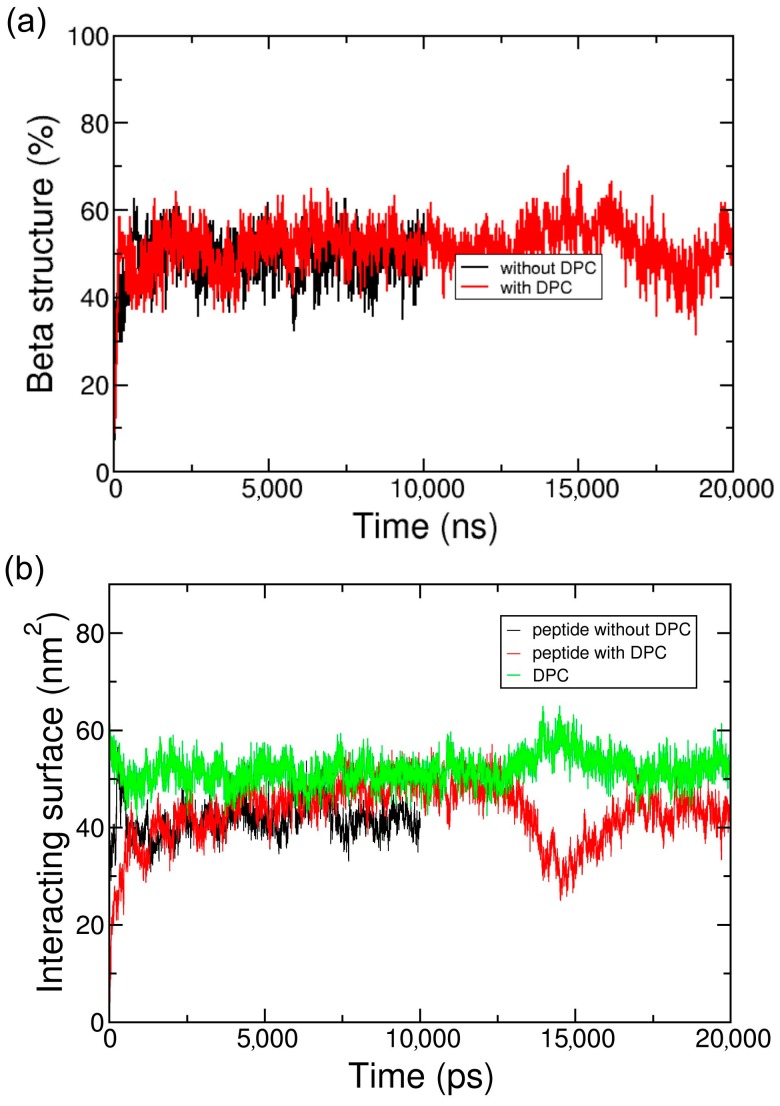
Secondary structure evolution (**a**) and surface of the interaction (**b**) of the peptide ADA8 in the presence of a membrane protein with (red lines) and without (black lines) DPC. The surface of the interaction between DPC and the membrane protein in the presence of the ADA8 peptide is in green.

ADA8 was shown by Tao et al. to be a very efficient peptergent and solubilize membrane proteins such as rhodopsin [13]. Thus, we chose an IMP with a known and well-characterized 3D structure, bacteriorhodopsin (BRD). Twenty peptides were simulated in water in the presence of one BRD protein over 10 µs in CG representation. Figure 2 and Figure S4a represent the peptides interacting with BRD at the end of the simulations; they show that the peptides form amphipathic beta sheets at the hydrophobic domain of the IMP, and present their hydrophilic residues to water. This process can be followed in Figure 3a and Figure S5a, which show an increase in the beta sheet content during the simulations, and Figure 3b and Figure S5b, where an increase in the area of the interacting surfaces between the peptides and the protein is observed. The peptides are rapidly attracted by the protein surface, and through interactions with BRD, they bury their hydrophobic residues. They form hydrophobic, polar, and backbone interactions between the antiparallel or parallel beta strands (Figure 2). Once at the protein surface, the reorganization of the peptides slowly occurs. The protein surface is 115 nm^2^, and the portion covered by the peptides represents 40 nm^2^. The peptides are also positioned mainly on the hydrophobic part of the protein. They are not always oriented parallel to the helices axis, which is expected for a beta barrel-like organization.

Figure 2c and Figure S4c show that when a detergent such as dodecylphosphocholine (DPC), is present at the surface of the protein, the peptide is able to go to the protein surface and form beta sheet structures similar to the situation without DPC. Furthermore, as the peptide is located on the transmembrane domain of BRD, it displaces DPC molecules from the hydrophobic core of the protein (Figure 2c). DPC molecules are still in interaction with BRD, notably on one of the apical regions where it can interact with two tyrosines and one phenylalanine residue (data not shown). As for the system without DPC, Figure 3 depicts the beta structure (Figure 3a and Figure S5a) and the surface of the interaction between the peptides and the protein (Figure 3b and Figure S5b) that are relatively stable along the simulations. To further assess the stability of the interactions and beta structure formed, the structures represented on Figure 2a,c have been transformed to an atomistic resolution and further simulated for 100 ns. These simulations show that the beta sheets formed in CG correspond to stable beta sheets in AT that keep their interactions at the protein surface (Figure S6).

### 2.3. Coarse-Grained Simulation of the Designed ABZ12 Peptide in the Presence of a Membrane Protein

**Figure 4:**
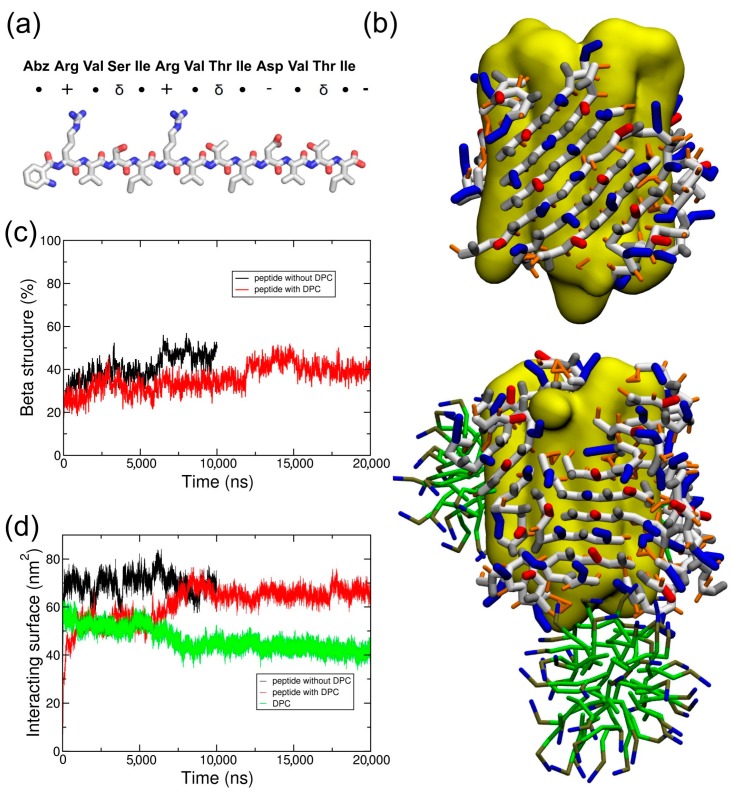
ABZ12 peptide structure and organization on the surface of the membrane protein. (**a**) Representation of the ABZ12 peptide. ●, δ, +, and - represent hydrophobic, polar, positively, and negatively charged residues, respectively; (**b**) The structures show 20 peptides at the end of the CG simulations when the protein (in yellow) is alone or covered by DPC (in green); (**c**) Secondary structure evolution and (**d**) surface of the interaction of the peptide ABZ12 in the presence of a membrane protein with (red lines) and without (black lines) DPC. The surface of the interaction between DPC and the membrane protein in the presence of the ABZ12 peptide is in green.

To test if this behavior could be extended to similar beta amphipathic peptides, we designed a peptide called ABZ12 (Figure 4a). To favor a β conformation, it is composed of residues that are most frequently found in β structures, such as ARG, VAL, ILE, or THR [22,23]. A size of 12 amino acids is also compatible with the width of the membrane bilayers, and is usually observed for membrane proteins with a beta barrel fold [24,25]. Hydrophobic amino acids (VAL and ILE) alternate with hydrophilic residues (ARG, THR, and SER) to generate amphipathy and promote the formation of beta strands [4]. Positive charges in the N-terminal part combined with negative charges at the C-terminal part should allow an antiparallel arrangement while keeping a global neutrality. A fluorescent N-terminal cap is added in the form of an aminobenzoyl group for experimental purposes. Infrared spectroscopy assays of the peptide (Figure S3) show a peak at approximately 1630 cm^−1^, which is characteristic of β-sheet conformations. According to what was carried out for ADA8, CG simulations of the system BRD/ABZ12 in the presence or absence of DPC molecules were calculated. As shown on Figure 4b, the same molecular picture is obtained; ABZ12 forms a beta belt around the membrane protein and displaces detergent molecules when they are present, suggesting a “peptergent-like” behavior. Actually, the detergents moved to more apical regions of the protein in the presence of the peptides. The beta structure of the peptides stays stable along the simulations (Figure 4c), and the contact surface between DPC and the protein decreased by at least 10 nm^2^ (Figure 4d). For the ABZ12 peptide, the formation of DPC micelles can be observed.

## 3. Discussion

In this study, we have analyzed the molecular behavior of ADA8, a well-described peptergent, for the solubilization and stabilization of IMPs through the formation of amphipathic beta barrel structures by molecular dynamic simulations. The peptide self-assembles into beta structures in water, and is able to interact with membrane proteins, which is in agreement with the experimental data previously published [13]. In water, the peptide forms amphipathic beta sheets that look like β-barrel for the AT representation, or “sandwich” like β-sheets. It is worth noting that the peptides were successfully simulated in atomistic and coarse-grained representations, validating the CG approach for such amphipathic peptides. The validation of the CG approach was assessed by using reverse transformations: hence, AT simulations carried out after reverse transformation showed that the beta sheets formed in CG were still stable.

When a membrane protein is present, the peptide steadily forms a beta sheet structure at the protein surface, and is able to displace DPC surfactants. Tao et al. proposed a model for the organization of the peptides around an IMP [13]. The peptides were thought to generate a beta-barrel belt around the hydrophobic helical domain that could help stabilize purified membrane proteins. The MD approaches developed in our study rather agree with this view and provide further molecular details for the replacement of detergent molecules around the protein. Although a complete belt was not obtained during the course of the simulations, the system tended to move toward this configuration.

In their work, Tao et al. also asked how IMPs are stabilized by beta strand peptides that can assemble into beta-aggregated structures in solution [13]. Our calculations suggest that beta sheet formation is favored in water, suggesting a strong peptide–peptide interaction. In the presence of membrane proteins, even those solubilized with surfactants, the ADA8 peptide could also form beta sheets at the protein surface. As the peptide–peptide interaction is stronger than that of surfactants, the former appears to steadily displace surfactants from the protein hydrophobic surface. Since Tao et al. showed that their model membrane protein retains its activity [13], we assumed that the IMP structure is not restrained by the beta sheet structure. Our calculations further suggest that the belt formed around the membrane protein is not “perfect”. The beta strand can be parallel or antiparallel, and beta sheets that are perpendicular to the protein α helices are observed.

Our MD approach could be used to select and design peptides with “peptergency” properties, i.e., amphiphilic peptides with β sheet structure propensity and the ability to form a β belt-like structure around an IMP in the presence or absence of detergent molecules. As an example, we have designed an ABZ12 peptide with a β conformation. As for ADA8 and as expected, this peptide is able to surround the membrane protein and displace the surfactant molecules to the more apical region of the IMP. The preliminary results of fluorescence resonance energy transfer (FRET) assays with ABZ12 show an energy transfer between the IMP and the aminobenzoic acid group of ABZ12 (data not shown), suggesting a direct interaction between ABZ12 and the protein. Future experimental investigations of the ability of ABZ12 to solubilize membrane proteins should help to confirm its “peptergency” potential.

In conclusion, our MD approach using atomistic and coarse-grained representations suggests that one possible mechanism for membrane proteins to be solubilized by β amphipathic self-assembling peptides is the formation of a belt-like structure around the IMP. This belt is not a perfect β barrel, modulating the view that was suggested previously [13]. To our best knowledge, this is the first molecular mechanism proposed for “peptergency”.

## 4. Materials and Methods

### 4.1. Peptide Synthesis

The ABZ12 peptide was synthesized by conventional solid phase peptide synthesis using Fmoc for transient NH_2_-terminal protection and was characterized using mass spectrometry. The peptide was lyophilized and resolubilized in DMSO at a final concentration of 10% (*w*/*v*) peptide as a stock solution. Before mixing with water, the peptide solution in DMSO was first diluted to 0.5% to avoid insolubility.

### 4.2. Fourier Transform Infrared (FTIR) Experiments

The infrared spectra were measured using a Bruker Equinox 55 spectrometer (Karlsruhe, Germany) equipped with a liquid nitrogen-cooled DTGS detector. The spectra were recorded from 4000 to 750 cm^−1^ in ATR (Attenuated Total Reflection) mode after 1024 scans at 4 cm^−1^ resolution, and at a two-level zero filling. During data acquisition, the spectrometer was continuously purged with filtered, dried nitrogen. For sample measurement, the peptide solubilized in DMSO was deposited on a germanium plate, and DMSO was evaporated under a N_2_ flux for approximately 5 h. The reference spectra of the germanium plate were automatically recorded and subtracted from the sample spectrum. The resulting spectrum was then smoothed using the Savitzky–Golay algorithm available in the OPUS software (Bruker, Germany).

### 4.3. Systems Studied

Two peptides were studied by molecular dynamic (MD) simulations; their properties are depicted in Figure 1a and Figure 4a. The ADA8 peptide is described in Tao et al. [13]; it contains two non-natural 2-aminodecanoic acids (ADA) and is acetylated at the N-terminus and amidated at the C-terminus (Figure 1a) [13]. The simulated peptide was not *N*-methylated. This peptide is barely soluble in aqueous solutions, and in their study, Tao et al. added *N*-methyl substituents to increase its solubility [13]. The ABZ12 peptide was designed for this study; it is capped at the N-terminus by an aminobenzoic acid, and is free at the C-terminus. The peptides have been modeled in an extended conformation based on experimental evidence. The force field Gromos96 54a7 (G54a7) [26] was used during this study. The ABZ topology came from a study by Song et al. in 2010 [27], and the ADA topology was derived from the ILE amino acid. The SPC model [28] was used to simulate water. The MARTINI force field [17,29] has been used for coarse-grained (CG) simulations. The ABZ residue was replaced by a PHE residue during the coarse-grained simulations. The membrane protein used was bacteriorhodopsin (PDBID: 1PY6), and its tertiary structure has been maintained with the SAHBNET network [30].

### 4.4. Atomistic Molecular Dynamic Simulations

Simulations were performed with the G54a7 force field [26]. All of the systems studied (see Supplementary Materials Table S1) were first minimized by steepest descent for 5000 steps. Then, a 1-ns simulation with the peptides under position restraints was run before the production simulations were performed. Periodic boundary conditions (PBC) were used with a 2-fs time step. All of the systems were solvated with SPC water [28], and Na^+^ ions were then added to neutralize the systems. The dynamics were carried out under NPT conditions (298 K and 1 bar). The temperature was maintained using the v-rescale method [31] with *τ*T = 0.1 ps, and an isotropic pressure was maintained using the Parrinello–Rahman barostat [32] with a compressibility of 4.5 × 10^5^ (1/bar) and *τ*P = 5 ps. The non-bonded interactions were evaluated using a twin-range method. Interactions within the short-range cutoff of 0.8 nm were calculated at every step. Interactions within the long-range cutoff of 1.4 nm were recalculated every 10 steps, together with the pair list. To correct for the truncation of electrostatic interactions beyond the long-range cutoff, a reaction-field correction was applied using a value of 61 for the relative dielectric permittivity [33]. Bond lengths were maintained with the LINCS algorithm [34]. Trajectories were performed and analyzed with GROMACS 4.5.4 tools as well as with homemade scripts and software. MDAnalysis was also used [35]. The 3D structures were analyzed with both the PyMOL [36] and VMD [37] softwares. The secondary structures were computed with STRIDE [38].

### 4.5. Coarse Grained Molecular Dynamic Simulations

The peptide models and the BRD protein were converted to a CG representation that was suitable for the MARTINI force field [17] with the martinize script [39]. Parameters have been developed for the ADA residue from the LEU parameters and atomistic simulations, and were added to the martinize script (topologies can be found in the supplementary files). No secondary structure was assigned to the peptides through dihedrals, and protections of the N-termini and C-termini were taken into account by setting the first and last BB beads to the P5 type. The coarse-grained peptides were placed in a simulation box with water (see Table S1). A total of 5000 steps of steepest-descent energy minimization was performed to remove any steric clashes, and production simulations were run. Temperature and pressure were set at 298 K and 1 bar using the weak coupling Berendsen algorithm [40] with *τ*T = 1 ps and *τ*P = 1 ps. Pressure was coupled isotropically. Non-bonded interactions were computed up to 1.2 nm with the shift method. Electrostatic interactions were treated with *ε* = 15. The compressibility was 3 × 10^4^ (1/bar). Coarse-grained simulations were carried out using GROMACS 4.5.4 [41].

To compare the structure evolution between AT and CG, we had to compute a parameter representing the beta structure in CG. Hence, as the backbone is only represented by one bead in CG, it is not possible to compute the phi/psi angles. A dihedral angle greater than 100° and the proximity of two other bonded backbone beads within 6 Å are used to consider a bead to be part of a beta sheet structure. These values have been taken from atomistic simulations, and allow for the calculation of beta structure content with enough precision (see Figure 1b). The interacting surface between peptides or DPC with the BRD protein has been computed by using the GROMACS sasa tool with a probe radius of 0.256 nm, which corresponds to the radius of the MARTINI particles. The interacting surface correspond to (SASA_prot_ + SASA_pep_ − SASA_prot+pep_)/2.

Backwards is used for the reverse transformation from a coarse-grained to an atomistic representation [42]. The ADA8 peptide is first transformed to a version of the peptide with neutral termini and without the N-terminal and C-terminal protections. The missing atoms are then added with an in-house script. A mapping file has been created for the ADA residue.

## Supplementary Materials

Supplementary materials can be found at http://www.mdpi.com/1422-0067/19/9/2772/s1.
