# Supplementary material for: Insight into the Self-Assembling Properties of Peptergents: A Molecular Dynamics Simulation Study
Source: Int J Mol Sci. 2018 Sep 14;19(9):2772. doi: 10.3390/ijms19092772 (PMC6163580; doi:10.3390/ijms19092772)
Supplement: Supplementary file 1 [file ijms-19-02772-s001.pdf]

International Journal of
Molecular Sciences

MDPI

Table S1. Atomistic and coarse-grained simulations carried out for the ADA8 peptide. Simulations CG3 and CG6 have also been carried out for the ABZ12 peptide.

Run	System	Box size (nm)	Simulation time (ns)
AT1	$Peptide_1/Water_{3097}/Na^+_2$	5.1 x 5.1 x 3.6	3 x 50
AT2	$Peptide_{10}/Water_{8238}/Na^+_{20}$	6.4 x 6.4 x 6.4	3 x 1,000
CG1	$Peptide_1/Water_{289}/Na^+_{20}$	3.9 x 2.7 x 3.3	3 x 10
CG2	$Peptide_{10}/Water_{7698}/Na^+_{20}$	10.0 x 10.0 x 10.0	3 x 10,000
CG3	BRD[1]/$Peptide_{20}/Water_{7045}/Na^+_{40}$	10.0 x10.0 x 10.0	3 x 10,000
CG4	BRD[1]/$DPC_{150}/Water_{6518}$	10.0 x 10.0 x 10.0	3,000
CG5	BRD[1]/$DPC_{104}/Water_{4852}$	9.0 x 9.0 x 9.0	1,000
CG6	BRD[1]/$Peptide_{20}/DPC_{104}/Water_{12666}/Na^+_{40}$	11.0 x 11.0 x 11.0	3 x 20,000

[1] BRD= bacteriorhodopsin protein

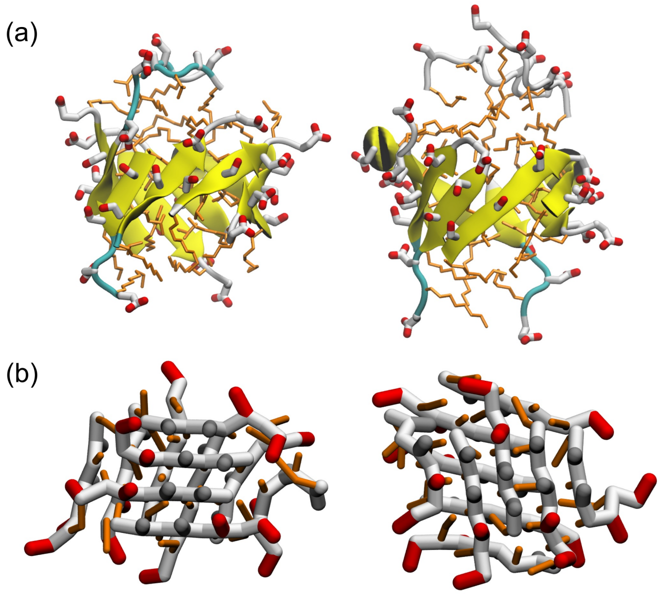

Figure S1. ADA8 peptides structure in water for the two other MD simulation repeats. Conformations at the end of simulations in atomistic and coarse grained representations are in panels (a) and (b), respectively. AT beta sheets are in yellow in the AT representations. Polar, negatively charged and hydrophobic CG residues are represented in gray, red and orange, respectively.

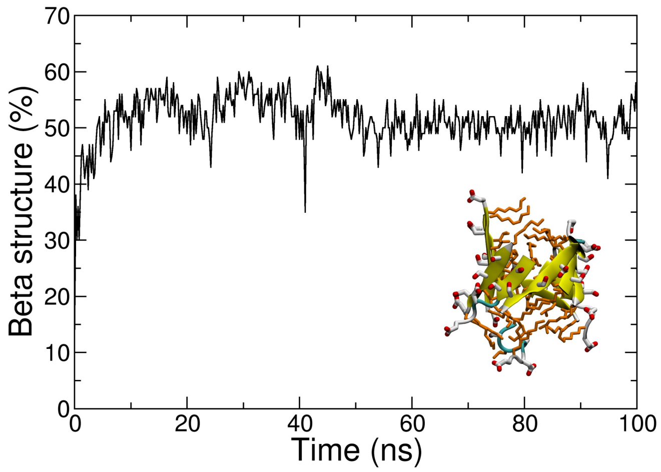

Figure S2. The secondary structure evolution of the ADA8 peptide after a reverse transformation from coarse grained to atomistic resolution. Insets show the structures at the end of the simulations.

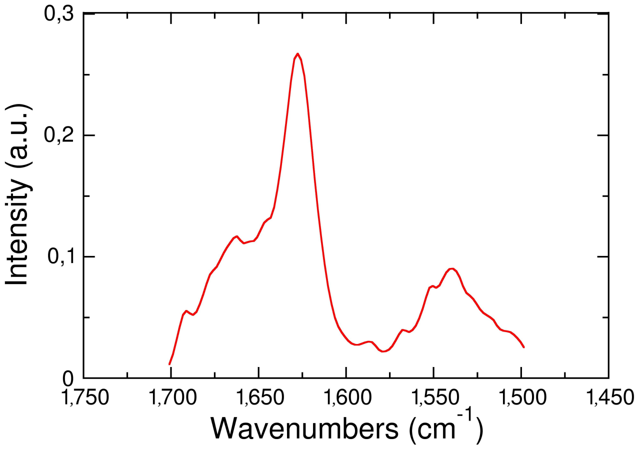

Figure S3. ATR-FTIR spectrum of the amide I band of the ABZ12 peptide. 200 µg of peptides have been dissolved at 1% weight/volume in DMSO.

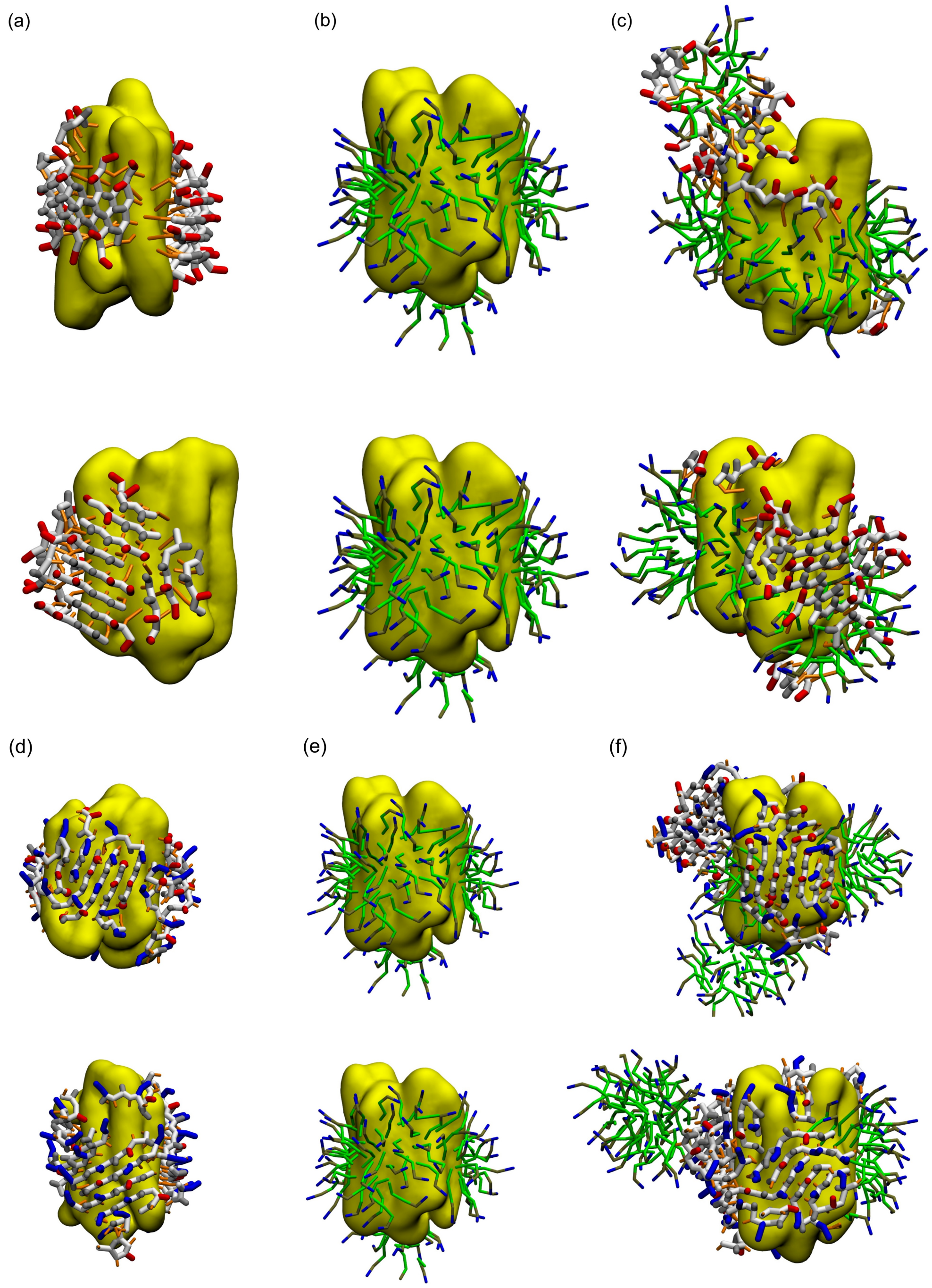

Figure S4. ADA8 (a,c) and ABZ12 (d,f) peptide organization on the surface of the membrane protein for the two other MD simulation repeats. The structures show 20 peptides at the end of the CG simulations when the protein (in yellow) is alone (a,d) or covered by DPC (c,f). The protein in a micelle of DPC is shown (b,e), and this structure was used as a starting point before the addition of the peptides (c,f).

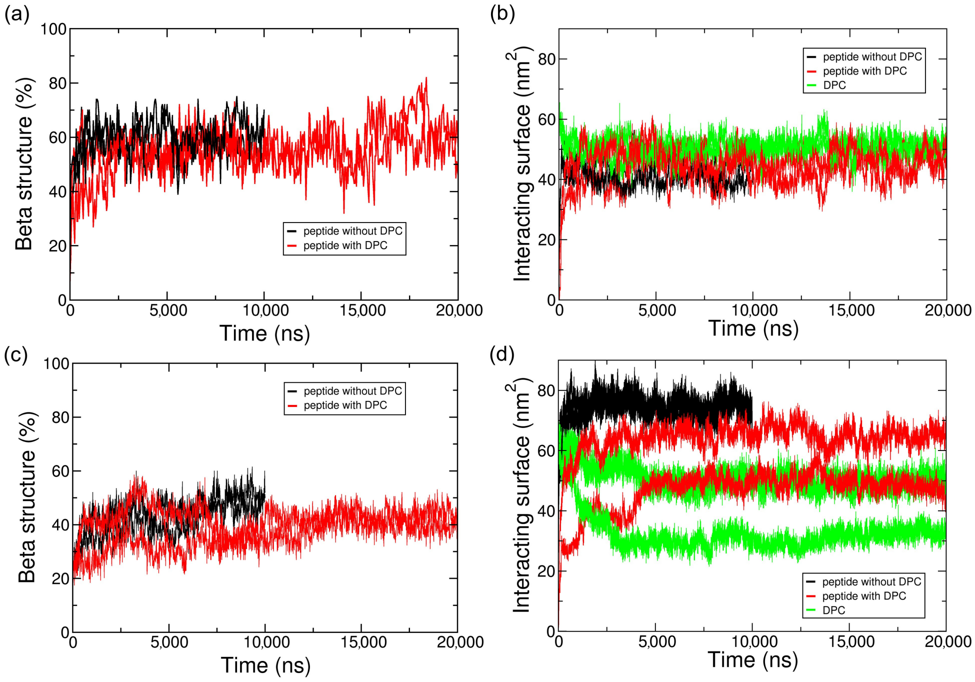

Figure S5. The secondary structure evolution (a,c) and surface of the interaction (b,d) of the peptides ADA8 (a,b) and ABZ12 (c,d) in the presence of a membrane protein with (red lines) and without (black lines) DPC. The surface of the interaction between DPC and the membrane protein in the presence of the ABZ12 peptide is in green.

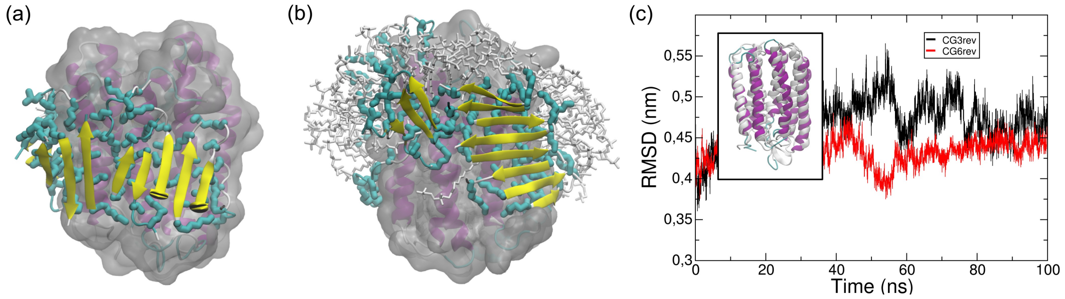

Figure S6. Structures after coarse grained to atomistic transformation and 100 ns of MD simulation. Initial structures are taken from simulations CG3 (**a**) and CG6 (**b**); BRD helices are in purple and the protein surface in grey, ADA8 beta strands are in yellow and its hydrophobic residues in cyan, and DPC molecules are in white sticks. (**c**) RMSD evolution for the BRD protein and structural alignment with the PDB structure.
